# Biomechanical effects of hemin and sildenafil treatments on the aortic wall of chronic-hypoxic lambs

**DOI:** 10.3389/fbioe.2024.1406214

**Published:** 2024-07-03

**Authors:** Álvaro Navarrete, Matías Inostroza, Andrés Utrera, Alejandro Bezmalinovic, Alejandro González-Candia, Eugenio Rivera, Carlos Godoy-Guzmán, Emilio A. Herrera, Claudio García-Herrera

**Affiliations:** ^1^ Laboratorio de Biomecánica y Biomateriales, Departamento de Ingeniería Mecánica, Universidad de Santiago de Chile, USACH, Santiago de Chile, Chile; ^2^ Institute of Health Sciences, University of O’Higgins, Rancagua, Chile; ^3^ Laboratorio de Ingeniería de Tejidos, Centro de Investigación Biomédica y Aplicada (CIBAP), Escuela de Medicina, Universidad de Santiago de Chile, Santiago de Chile, Chile; ^4^ Pathophysiology Program, Institute of Biomedical Sciences (ICBM), Faculty of Medicine, Universidad de Chile, Santiago, Chile; ^5^ International Center for Andean Studies (INCAS), Universidad de Chile, Santiago, Chile

**Keywords:** chronic hypoxia, artery wall, hemin, sildenafil, pre-stretching test, ring-opening test, uniaxial-tensile test

## Abstract

**Introduction:** Gestation under chronic hypoxia causes pulmonary hypertension, cardiovascular remodeling, and increased aortic stiffness in the offspring. To mitigate the neonatal cardiovascular risk, pharmacological treatments (such as hemin and sildenafil) have been proposed to improve pulmonary vasodilation. However, little is known about the effects of these treatments on the aorta. Therefore, we studied the effect of hemin and sildenafil treatments in the aorta of lambs gestated and raised at highlands, thereby subjected to chronic hypoxia.

**Methods:** Several biomechanical tests were conducted in the descending thoracic aorta (DTA) and the distal abdominal aorta (DAA), assessing 3 groups of study of hypoxic animals: non-treated (Control) and treated either with hemin or sildenafil. Based on them, the stiffness level has been quantified in both zones, along with the physiological strain in the unloaded aortic duct. Furthermore, a morphological study by histology was conducted in the DTA.

**Results:** Biomechanical results indicate that treatments trigger an increment of axial pre-stress and circumferential residual stress levels in DTA and DAA of lambs exposed to high-altitude chronic hypoxia, which reveals a vasodilatation improvement along with an anti-hypertensive response under this characteristic environmental condition. In addition, histological findings do not reveal significant differences in either structure or microstructural content.

**Discussion:** The biomechanics approach emerges as a valuable study perspective, providing insights to explain the physiological mechanisms of vascular function. According to established results, alterations in the function of the aortic wall may not necessarily be explained by morphostructural changes, but rather by the characteristic mechanical state of the microstructural components that are part of the studied tissue. In this sense, the reported biomechanical changes are beneficial in mitigating the adverse effects of hypobaric hypoxia exposure during gestation and early postnatal life.

## 1 Introduction

High-altitude hypobaric hypoxia (HAHH) corresponds to a drop in the atmospheric partial pressure of oxygen $\left({PO}_{2}\right)$ , a condition that naturally occurs in highlands. This environmental condition leads to a decrease in the partial pressure of oxygen on arterial blood $\left({PaO}_{2}\right)$ relative to normoxic-normobaric conditions (exposure to lowlands) (Brito et al., 2020; Navarrete et al., 2020). This is clinically relevant at altitudes higher than 2,500 m-above-sea-level (m.a.s.l.), where pathophysiological responses become evident. Chronic exposure to HAHH during gestation may lead to perinatal complications, such as fetal hypoxia, respiratory distress, pulmonary arterial hypertension and aortic dysfuntion, among others (Gonzaléz-Candia et al., 2020; González-Candia and Herrera, 2021), specifically in the transition between fetal to neonatal circulation. This type of cardiovascular disease is related to an increment of vasoconstriction generated by smooth-muscle (Touyz et al., 2018; Jaminon et al., 2019), due to a pathological structural remodelling in small pulmonary arteries. A significant fraction of the world’s population lives under the effects of HAHH (between 80 and 140 million people) (Herrera et al., 2015; Tremblay and Ainslie, 2021), so the interest in studying its possible negative effects has a relevant contemporary concern in many disciplines.

In the area of biological soft-tissue characterization, only a small number of research studies have been focused on determining the *ex-vivo* mechanical response of the artery wall in face of cardiovascular diseases (Pejcic et al., 2019), such as atherosclerosis (Walsh et al., 2014; Karimi et al., 2015), abdominal aortic aneurysms (O’Leary et al., 2014; Pancheri et al., 2017) and vascular repair processes after stent implantation (Evans and Kwak, 2013; Fortier et al., 2014). In specific, the effects of chronic exposure to HAHH on the mechanical properties of arteries have been only barely explored. Drexler et al. (2008) reported stiffening on extrapulmonary arteries (trunk, right, and left main arteries) of rats with pulmonary hypertension induced by high altitude (5,000 m). In a recent experimental study, Utrera et al. (2022) described mechanical and structural alterations on descending thoracic aortas of rats as a cycle-dependent effect of the exposure to intermittent HAHH.

Treatments for pulmonary hypertension have been in the spotlight for the last 20 years, mainly aimed at reducing the intense vasoconstriction, the risks of blood-clots and on increasing pulmonary vasodilatation (Navarrete et al., 2020). Several studies focused on vascular reactivity have assessed the influence of a melatonin treatment and its effect on chronic HAHH (Beñaldo et al., 2019; Figueroa et al., 2021; Gonzaléz-Candia et al., 2021). From a biomechanical point of view, the performance of different treatments applied in the aforementioned context on the passive mechanical response of arteries, has not been widely assessed. Rivera et al. (2020) studied the effect of melatonin on thoracic, main pulmonary and abdominal aortic arteries of lambs subjected to chronic HAHH exposure. Their main results did not reveal significant evidence of changes on the *ex-vivo* mechanical response of arterial tissue. A similar trend was reported by Bezmalinovic et al. (2021), who addressed the hyperelastic and damage properties of thoracic aortas in the same animal model and treatment as Rivera et al. (2020). Besides, Navarrete et al. (2020) determined the effect of Atrial Natriuretic Peptide (ANP) and Cinaciguat on arterial residual strains, considering the same environmental conditions and animal model than Rivera et al. (2020). This study was performed on aorta, carotid and femoral arteries, finding significant changes in the residual strain measurements on treated subjects. More recently, Laubrie et al. (2023) studied the effect of Cinaciguat on the hyperelastic, damage and dissipation behavior of thoracic aortas on the same animal model as Rivera et al. (2020), Navarrete et al. (2020) and Bezmalinovic et al. (2021). The initial progress in this area reflects the need to better understand the effects of new treatments on the arterial-wall response, in order to have a more global overview related to the application of these therapies. Hemin is an inducer and activator of the heme oxygenase 1 enzyme (HO-1), which has inhibitory effects on the progression of pulmonary hypertension (Shimzu et al., 2008) due to its vasodilator, antioxidant and anti-remodeling properties. Sildenafil is a potent competitive inhibitor of phosphodiesterase type-5 (PDE5), which enhances the bioavailability of cyclic guanosine monophosphate (cGMP), resulting in vasodilation, which has already been proved as a successful treatment for reverting the effects of pulmonary hypertension (Weimann et al., 2000; Herrera et al., 2008). Both drugs are approved for treatments in humans, but only sildenafil has been used in neonates for cardiopulmonary problems, such as pulmonary hypertension and broncopulmonary dysplasia (Dillon et al., 2023). Sildenafil was proposed as a potent vasodilator agent (Kelly et al., 2017), while Hemin as an antiremodeling and antioxidant (Estarreja et al., 2024) In this context, there is strong evidence that suggests a potentially beneficial outcome of using these two drugs in treatments to reverse the adverse effects of HAHH exposure. However, to the best of our knowledge, no studies have addressed the effects of hemin and sildenafil on neonatal aortas exposed to chronic hypoxia during the perinatal period.

According to the different aspects previously exposed, the scope of this work is to determine the biomechanical response of thoracic and abdominal aorta arteries from lambs exposed to chronic HAHH and treated with either hemin or sildenafil, relating it to morphometric measurements. Specifically, mechanical properties obtained from uniaxial-tensile tests (along the circumferential and longitudinal directions of the arterial duct) and deformation measurements from residual strain tests − namely, ring-opening (García-Herrera et al., 2016) and pre-stretching tests (Voňavková and Horny, 2020) − are considered to assess the biomechanical impact of these treatments.

## 2 Material and methods

The Bioethics Committee of Universidad de Chile approved all experimental procedures (Protocol CBA 761 FMUCH). The study was performed according to the Guide for the Care and Use of Laboratory Animals published by the United States National Institutes of Health (NIH Publication No. 85–23, revised 1996) and adheres to the American Physiological Society’s Guiding Principles in the Care and Use of Animals.

### 2.1 Animals and treatments

Aortas were obtained from eighteen lambs (*Ovis aries*) aged 30 days-old, all of them gestated, born and raised at a high altitude (INCAS Research Station, Putre, 3,600 m. a.s.l). The use of this particular animal model is justified by several reasons, referred mainly to the cardiovascular development, structure, function and responses, similar to those observed in humans (Morrison et al., 2018; Gonzaléz-Candia et al., 2020; Navarrete et al., 2020).

Animals were randomly separated into three groups according to the applied treatment, as follows: Control group ( N=8 ; vehicle); Hemin group ( N=5 ; 10 mg/kg/24 h of hemin in NaOH 0.01N, SC, during 10 days); Sildenafil group ( N=5 ; 0.5 mg/kg/12 h of sildenafil in NaCl 0.9%, EV, during 5 days). In this context, to achieve a proper materno-neonatal attachment (at the beginning of *postpartum* period), all treatments were sumministrated since 3 days old. The doses were adjusted based on previous studies (Herrera et al., 2008; Dillon et al., 2023). In addition, before the experiments, we perform *in-vivo* dose response to ensure a pulmonary vascular response without any hemodynamic systemic responses (unpublished data).

### 2.2 Tested arterial tissue

**FIGURE 1:**
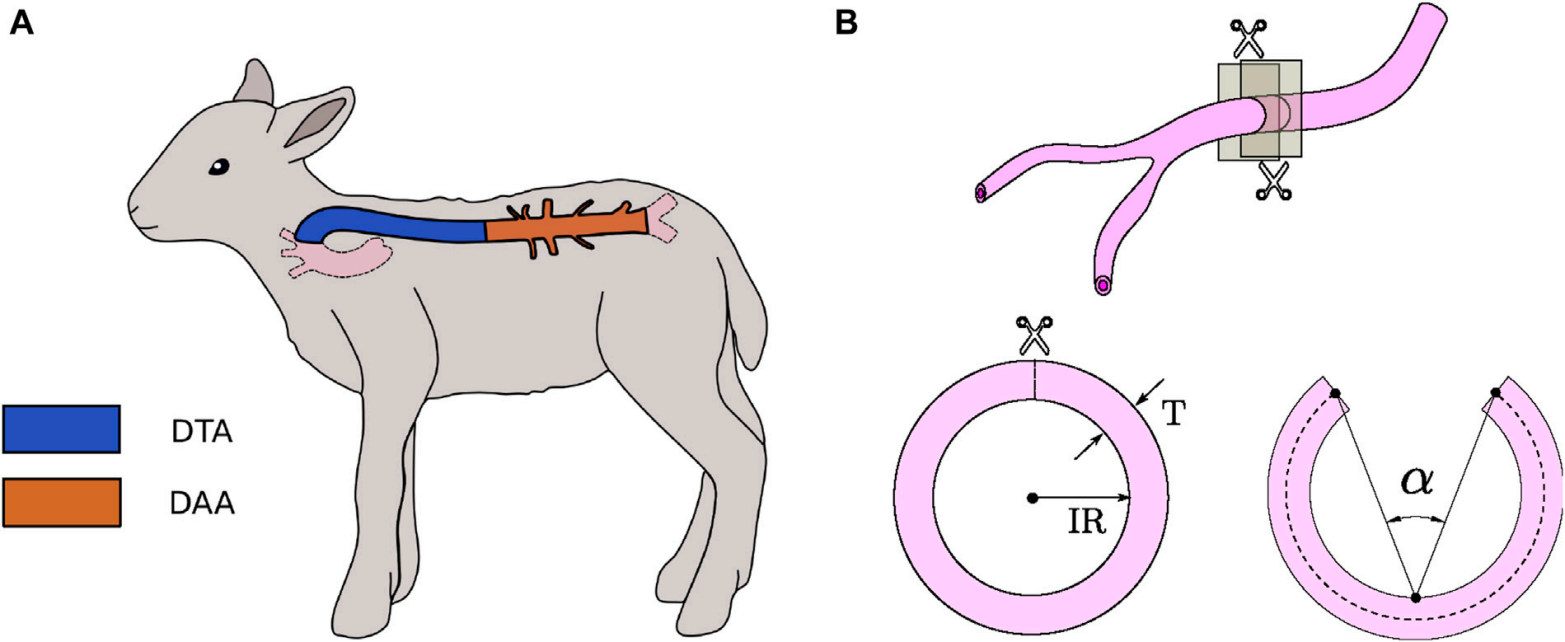
**(A)** Spatial localization of DTA and DAA artery regions in a sheep body. **(B)** Characteristic dimensions of closed and opened artery ring specimens.

Each aorta artery was divided into two regions, according to its location in the body (Figure 1A). The first region, called descending thoracic aorta (DTA, blue colored in Figure 1A), corresponds to the segment that goes from the end of the aortic arch to the diaphragm. The second segment, called distal abdominal aorta (DAA, brown colored in Figure 1A), is considered between the diaphragm and the right renal artery.

All protocols for tissue manipulation were carried out in the basis of the established by Navarrete et al. (2020). Immediately after euthanasia, initial measurements were performed on the animal body (see details in Section 2.3). Then, the artery was extracted and kept on a calcium-free saline solution (Krebs ${Ca}^{2+}$ -free) during the remaining *ex-vivo* measurements and the corresponding biomechanical tests.

### 2.3 Experimental procedure

The experimental procedure performed in this study is focused on determining potential similarities or differences in structure, composition, and mechanical response along the aortic region.

#### 2.3.1 Morphology

To determine the overall structure of the artery wall, morphologic measurements were carried out. The procedure consists on obtaining a ring-shaped sample from the excised artery, and place it on a Petri dish exposing its sectional area. Then, a photography is taken and analyzed with the aid of ImageJ software, which allows to scale the pixels of the image to an appropriate unit of measurement. So that, inner and outer perimeter can be obtained.

Two characteristic parameters of the vessel cross-section were defined, schematically represented in Figure 1B. The inner radius (IR) is determined by its inner perimeter, considering a circular-shaped cross-section for arteries. Concurrently, wall thickness (T) is quantified by the difference between outer radius (determined by the outer perimeter) and inner radius.

##### 2.3.1.1 Biomechanical tests

The biomechanical tests performed in this study were carried out according to protocols and procedures detailed in Navarrete et al. (2020), Rivera et al. (2020), and Utrera et al. (2022). The tests are briefly described below.

##### 2.3.1.2 Pre-stretching test

According to previous findings, it is known that arteries are subjected to a physiological longitudinal stretch (Guo et al., 2012), which accomplishes several physiological roles: it minimizes longitudinal stress in the artery wall, prevents buckling, and presents vasodilator effects by increasing circumferential distensibility Horny et al. (2017).

This phenomenon can be observed when comparing the *in-vivo* and *ex-vivo* lengths of an artery, where a non-homogeneous shortening is reported in different zones of the aorta (Navarrete et al., 2020; Rivera et al., 2020; Utrera et al., 2022). To quantify the shortening experienced along the artery on each aortic zone, six equidistant marks were drawn in the vessel, using a gel pencil. A photography is taken in the *in-vivo* configuration, and then the artery is extracted from the animal body and placed inside a Krebs-filled Petri dish. After 10 min, another photography is taken. A millimeter grid paper is placed near the vessel in both pictures as a reference for length measurement. Using ImageJ software, *in-vivo* and *ex-vivo* lengths ( $l_{\text{in}-\text{vivo}}$ and $l_{\text{ex}-\text{vivo}}$ respectively) are registered for each artery segment, and axial pre-stretch is computed by, $\lambda_{Z}=l_{\text{in}-\text{vivo}}/l_{\text{ex}-\text{vivo}}$ .

##### 2.3.1.3 Ring-opening test

The ring-opening test is a widely utilized method to determine circumferential residual strain, which allows to estimate indirectly the residual stress field on a blood vessel, whose physiological role consists of homogenizing the stress distribution in the arterial wall (García-Herrera et al., 2016).

Regarding the performed experimental procedure, once the artery was removed from the body and it was photographed for the pre-stretching test (Section 2.3.2), two 1 mm-long rings were obtained from the proximal and distal ends of the artery (DTA and DAA zones respectively). Ring samples were maintained in a Krebs ${Ca}^{2+}$ -free solution at a physiological temperature (39 ° C), and photographed 15 min later. Subsequently, a radial cut was performed on each ring, producing an abrupt change in geometry, then the sample was kept in the same solution for 15 more minutes in order to eliminate viscous effects on the material (Fung, 1991). Finally, opened rings were photographed, and its characteristic angle (α) – measured by considering (as rays) the lines formed by the points located at mid-thickness at the opened-ring ends (t/2) , and (as the vertex) the midpoint of the artery-wall perimeter at the inner radius (see Figure 1B) – is determined via image analysis with the aid of ImageJ software. Both closed and opened ring configurations were photographed using ToupView software under a magnifying glass (Motic SMZ-161) equipped with a digital camera (Motic Moticam 2.0MP.).

##### 2.3.1.4 Uniaxial-tensile test

In order to determine the Cauchy stress (σ) *versus* stretch (λ) response, two rectangular pieces of artery-wall were extracted from DTA (lower thoracic zone, above the diaphragm) and DAA (upper abdominal zone, below the diaphragm) zones of each animal. Also, their initial dimensions (width $w_{0}$ , length $l_{0}$ and thickness $t_{0}$ ) were measured, with values in between 3.3 and 7.5 mm for width, and values within 5 and 7.5 mm for length. The thickness was that of the artery itself. One of the samples was stretched along the longitudinal direction of the arterial duct, whereas the other was stretched along the circumferential direction. In both cases, the displacement-rate was set at 1.5 mm/min to minimize time-dependent effects on the material. The required instantaneous force (F) , reached at different levels of displacement (d) , was applied using a universal testing machine (Instron 3343) and captured using a 10 N load-cell ( ±0.001 N). Each test was carried out until the rupture of the specimen. During the entire test, samples were kept submerged in a Krebs ${Ca}^{2+}$ -free solution at a physiological temperature of 39 ° C.

Stretch (λ) was calculated as, $\lambda =\left(l_{0}+d\right)/l_{0}$ , and the assumption of incompressibility, usually considered for arterial tissue (Holzapfel, 2000), allows to compute Cauchy stress (σ) as, $\sigma =F\lambda /\left(w_{0}\;t_{0}\right)$ . Four mechanical parameters were determined from each stress-stretch curve, which allowed the quantification of the material stiffening at low and high stretches, along with the transition point between both strain levels (Utrera et al., 2022). From the first linear zone of the curve, the slope $E_{1}$ , corresponding to the elastic modulus at low-stretches, was determined. Likewise, a second linear zone at high-stretches was quantified by the elastic modulus $E_{2}$ . Finally, the transition point ( $\lambda_{\text{t}}$ , $\sigma_{\text{t}}$ ) was obtained, which denotes the boundary between low-stretch and high-stretch regimes of the tensile behavior. Both linear regions are identified at the moment of the slope analysis. The stretch bounds of each slope are hand-selected and manually visualized. A linear regression is calculated in the selected area, and the resulting slope is taken as the elastic modulus at either low or high strains. The determination coefficient is checked after every regression. If this coefficient does not reach a value near 1 (over 0.999), the boundaries of the analysis are changed until good fitness is achieved.

#### 2.3.3 Histology

**FIGURE 2:**
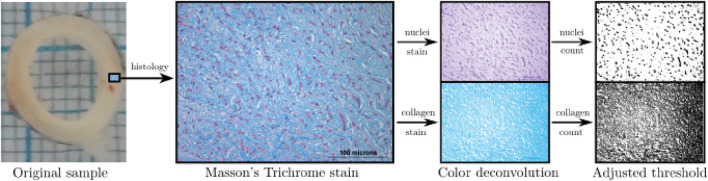
Protocol for histological measurements.

It is of utmost interest to quantify the composition of the DTA on the media layer, which mainly determines its elastic response. In this sense, a histological analysis was performed, following Masson’s Trichrome procedure (Leonard et al., 2018). Figure 2 shows a histological photograph, where blue zones denote the presence of collagen fibers whereas red/pink ones stain the cell nuclei.

From the Masson’s Trichrome staining, 4 images per group were taken of each cardinal point, following the procedure detailed in Navarrete et al. (2020). From each image, collagen percentage along with cell nuclei density were measured, and the mean value was taken as the representative of each animal. To measure the presence of collagen and cell nuclei, an image-processing procedure was carried out (see Figure 2), through the deconvolution technique and a subsequent application of a thresholding method (Utrera et al., 2022).

### 2.4 Statistical analysis

All values are expressed as mean ± SEM (standard error of the mean). The statistical analyses were carried out with Graphpad Prism 6.01 (GraphPad Software Inc., San Diego, CA, USA). Firstly, a Kolmogorov–Smirnov test was used to check normality of the datasets. Then, a F-test was performed to compare potential variance differences between groups. Once both statistical tests were performed, a suitable statistical method was chosen to determine mean differences. The unpaired t-test was performed in case of normal distribution of both groups and the same variance; the Welch t-test was applied in the case of normal distribution of both groups and statistically significant differences of variance between groups; and the non-parametric Mann-Whitney U test was performed when one or both groups did not show normality. All comparisons were performed among treated groups and between the treated groups and the Control group. Differences were considered statistically significant for a probability of the null hypothesis being true ( p -value), p≤0.05 . It should be noted that most of the groups analyzed have a normal distribution and statistically the same variances.

## 3 Results

### 3.1 Morphometry

To give a sense of the size in the DTA and DAA regions of arteries studied, the inner radius (IR) and thickness (T) of closed ring specimens (Figure 1B) from DTA and DAA zones, are exhibited in Table 1. No significant differences were found on the size of closed rings among experimental groups.

**TABLE 1:** Inner radius (IR) and thickness (T) of closed ring specimens extracted from DTA and DAA zones.

	Control		Hemin		Sildenafil	
	IR (mm)	T (mm)	IR (mm)	T (mm)	IR (mm)	T (mm)
DTA	3.28±0.16	2.27±0.11	3.09±0.24	2.47±0.17	3.44±0.09	2.04±0.13
DAA	2.39±0.13	1.07±0.05	2.44±0.23	1.07±0.04	2.71±0.17	0.97±0.05

Results for control (N = 8) and hemin/sildenafil (both with N = 5) treated groups. Values expressed as mean ± SEM. No significant differences (p≤0.05) were detected.

### 3.2 Biomechanical tests

#### 3.2.1 Pre-stretching test

**FIGURE 3:**
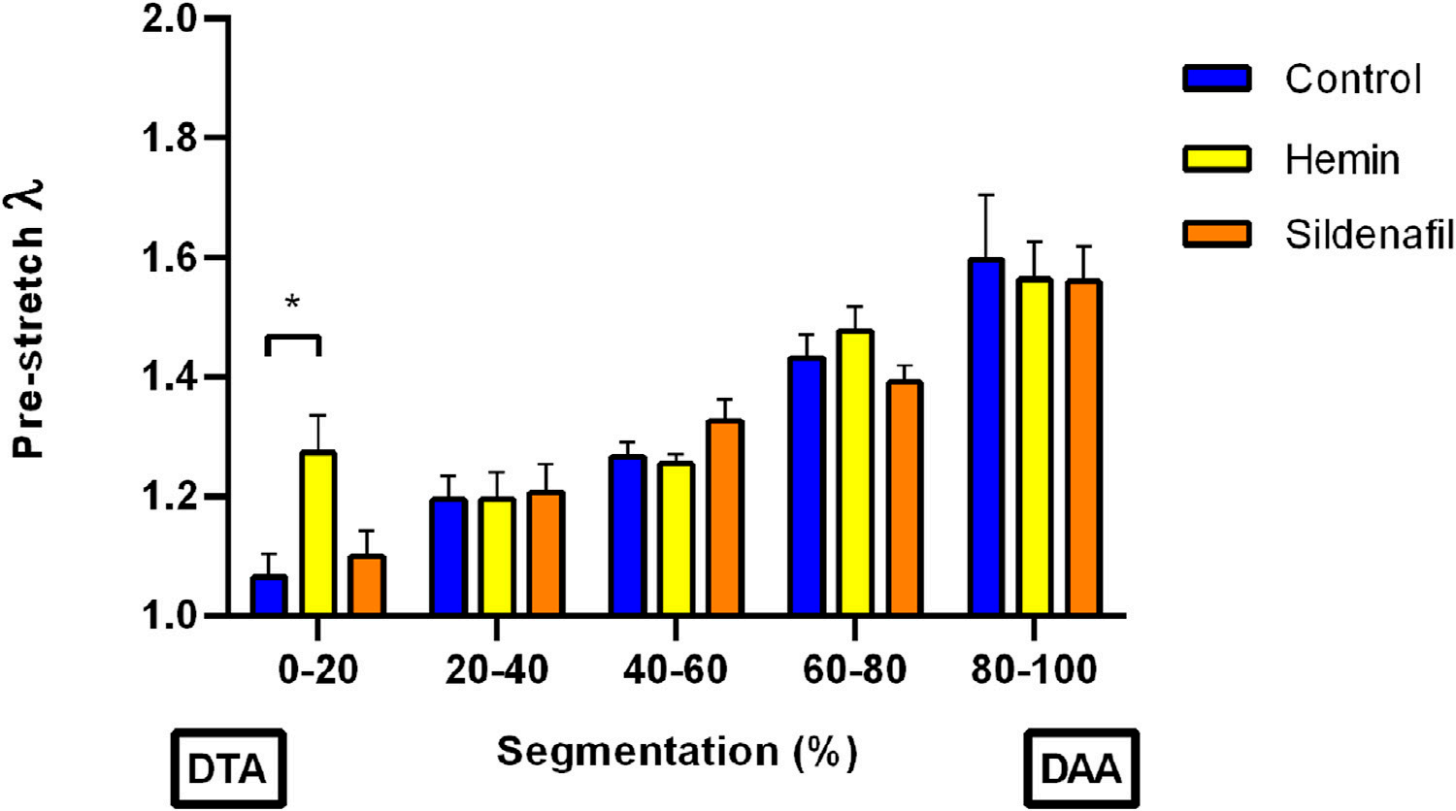
Pre-stretching test. Pre-stretch *versus* segmentation (location segments within the aorta), from descending-thoracic (DTA) to distal-abdominal (DAA) aorta regions of control (N = 8) and hemin/sildenafil (both with N = 5) treated groups. Values expressed as mean ± SEM. Significant differences (p≤0.05) : (*) vs. Control.

Figure 3 shows pre-stretch values $\left(\lambda_{Z}\right)$ for each region (DTA to DAA) of the aorta. Each specimen was divided into five sections of roughly equal relative length. According to general results, through statistical analysis (Section 2.4) it is possible to state that there is not interaction between segmentation and group effects (p=0.12) . Focusing on the variation of $\lambda_{Z}$ along the aorta of each study group, there is a characteristic incremental trend from DTA to DAA zones. In specific, results of the control group ranged from 1.07 ± 0.04 to 1.60 ± 0.11, whereas results of hemin group varied from 1.27 ± 0.06 to 1.56 ± 0.06, and sildenafil group exhibited a variation from 1.10 ± 0.04 to 1.56 ± 0.06.

A comparison of $\lambda_{Z}$ values on each aorta segment shows that most of them did not have a significant variation among groups. The only exception is found in the hemin-treated group, where in the proximal segment (0–20 % ) a higher average pre-stretch value is present with respect to the values of other groups in this same region. This effect is statistically significant when comparing it with the Control group.

#### 3.2.2 Ring-opening test

**FIGURE 4:**
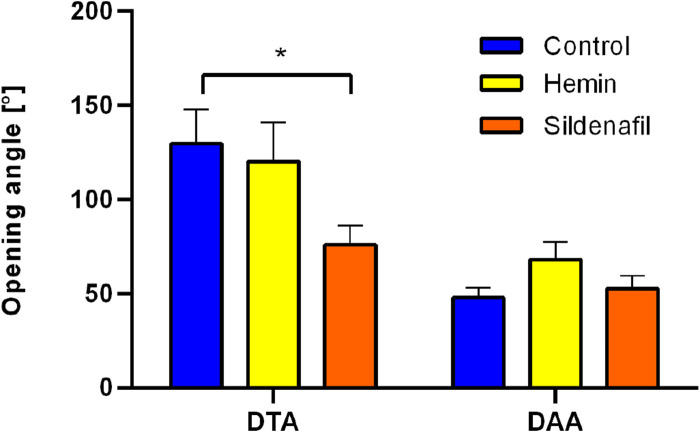
Ring-opening test. Opening angle of descending-thoracic (DTA) and distal-abdominal (DAA) aortas of control (N = 8) and hemin/sildenafil (both with N = 5) treated groups. Values expressed as mean ± SEM. Significant differences (p≤0.05) : (*) vs. Control.

Figure 4 shows opening-angle values of DTA and DAA zones for the different study groups. From the results, a marked difference between average values of both regions can be observed on all groups, with statistically-significant differences in the control group (DTA → 125.4 °± 13.3 ° vs. DAA → 45.7 °± 8.0 ° ) and hemin group (DTA → 115.5 °± 24.8 ° vs DAA → 50.2 °± 12.7 ° ). Regarding the comparison between treatments in a specific aorta region, a significant difference was found when contrasting the DTA zone of control and sildenafil groups (125.4 °± 13.3 ° vs 65.7 °± 9.1 ° , respectively).

#### 3.2.3 Uniaxial-tensile test

Tables 2, 3 show the mechanical parameters, which are defined in Section 2.3.2, determined from uniaxial stress-stretch curves of DTA and DAA zones, respectively. These results are presented for the Control and (hemin or sildenafil) treated groups, along longitudinal and circumferential directions of the vessel. The mean stress-stretch curves for both arteries and directions are displayed in the supplementary material.

**TABLE 2:** Uniaxial-tensile test. Parameters obtained from stress-stretch curves of descending thoracic aortas (DTA) of control (N = 8) and hemin/sildenafil (both with N = 5) treated groups: initial slope $E_{1}$ (at low-stretches), final slope $E_{2}$ (at high-stretches) and coordinates of the transition between low and high stretch regimes ( $\lambda_{\text{t}}$ , $\sigma_{\text{t}}$ ).

DTA	Control		Hemin		Sildenafil	
	Long.	Circ.	Long.	Circ.	Long.	Circ.
$E_{1}$ (kPa)	65.24 ± 13.89	58.10 ± 7.19	92.05 ± 35.61	85.49 ± 23.74	119.7 ± 12.9	129.7 ± 10.0*
$E_{2}$ (kPa)	204.1 ± 20.8	395.7 ± 96.5	256.9 ± 69.3	505.9 ± 85.9	279.1 ± 22.4	954.7 ± 106.9*
$\lambda_{\text{t}}$ (–)	1.36 ± 0.08	1.57 ± 0.07	1.61 ± 0.21	1.61 ± 0.07	1.52 ± 0.08	1.52 ± 0.02
$\sigma_{\text{t}}$ (kPa)	39.50 ± 15.17	63.61 ± 14.54	62.89 ± 33.17	80.36 ± 15.20	69.61 ± 9.80	113.5 ± 12.5

Results of tests along longitudinal (Long.) and circumferential (Circ.) directions. Values expressed as Mean ± SEM. (*) Significant differences (p≤0.05) : vs. Control.

**TABLE 3:** Uniaxial-tensile test. Parameters obtained from stress-stretch curves of distal abdominal aortas (DAA) of control (N = 8) and hemin/sildenafil (both with N = 5) treated groups: initial slope $E_{1}$ (at low-stretches), final slope $E_{2}$ (at high-stretches) and coordinates of the transition between low and high stretch regimes ( $\lambda_{\text{t}}$ , $\sigma_{\text{t}}$ ).

DAA	Control		Hemin		Sildenafil	
	Long.	Circ.	Long.	Circ.	Long.	Circ.
$E_{1}$ (kPa)	120.5 ± 18.4	126.1 ± 22.1	162.2 ± 33.5	295.6 ± 55.1	222.5 ± 33.0	361.7 ± 50.6*
$E_{2}$ (kPa)	1451 ± 378	1198 ± 265	1303 ± 284	2437 ± 451	2051 ± 498	2171 ± 483
$\lambda_{\text{t}}$ (–)	1.50 ± 0.09	1.41 ± 0.03	1.45 ± 0.04	1.48 ± 0.08	1.50 ± 0.07	1.49 ± 0.08
$\sigma_{\text{t}}$ (kPa)	156.9 ± 50.6	89.63 ± 18.31	128.7 ± 24.5	251.1 ± 63.4	230.1 ± 68.3	304.4 ± 81.6*

Results of tests along longitudinal (Long.) and circumferential (Circ.) directions. Values expressed as Mean ± SEM. (*) Significant differences (p≤0.05) : vs. Control.

From the results displayed in Tables 2, 3, no significant differences of mechanical parameters were found when comparing the hemin-treated and Control groups. On the other hand, according to Table 2, significant differences in the slope at low-stretches $E_{1}$ and at high-stretches $E_{2}$ can be observed between sildenafil and Control groups on samples tested along the circumferential direction. In specific, on DTA zones, the treatment increases both values, which denotes a higher uniaxial stiffness of the material at low and high stretches. Conversely, in Table 3, DAA zones of sildenafil treated subjects only show a higher $E_{1}$ , coherent with higher values of transition stress $\sigma_{\text{t}}$ , at similar transition stretch $\lambda_{\text{t}}$ .

### 3.3 Histology

**FIGURE 5:**
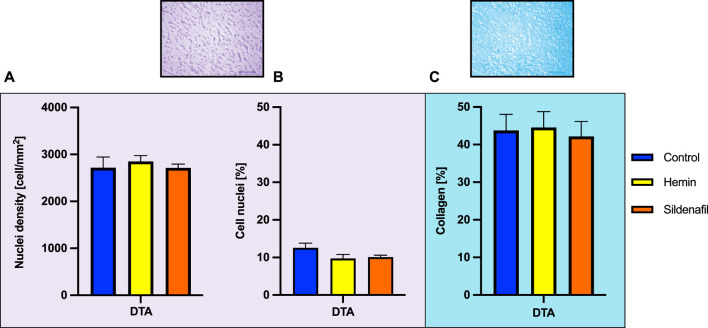
Histological measurements. Values obtained from histology of descending thoracic aorta (DTA) of control (N = 8) and hemin/sildenafil (both with N = 5) treated groups. **(A)** Cell nuclei density, **(B)** cell nuclei percentage, and **(C)** collagen percentage within the arterial wall. Values expressed as Mean ± SEM. Significant differences (p≤0.05) (*) vs. Control.

A crucial aspect of correlating the previously reported biomechanical changes with the microstructural composition of the artery wall involves the application of histological techniques, duly detailed in Section 2.3.3. Figure 5 exhibits the main results obtained from the information provided by the Masson’s Trichrome stain on the DTA: cell nuclei density (Figure 5A), percentage area covered by cell nuclei (Figure 5B), and collagen percentage (Figure 5C).

In any case, there were no significant differences between the non-treated group and the respective groups with treatment applied. Particularly, SMC nuclei density by each group was: Control → 2,718 ± 85 cells/ ${mm}^{2}$ , hemin → 2,852 ± 62 cells/ ${mm}^{2}$ , and sildenafil → 2,715 ± 35 cells/ ${mm}^{2}$ . The results for percentage area occupied by cell nuclei were the following: Control → 12.6 ± 1.2%, hemin → 9.7 ± 1.1%, and sildenafil → 10.1 ± 0.5%. Finally, the collagen percentage detailed by group was: Control → 43.75 ± 1.62%, hemin → 44.58 ± 2.09%, and sildenafil → 42.19 ± 1.78%.

## 4 Discussion

Exposure to chronic hypoxia during gestation leads to pulmonary hypertension, cardiovascular remodeling, and increased aortic stiffness in offspring (Herrera et al., 2010). To mitigate neonatal cardiovascular risks, potential pharmacological interventions such as hemin and sildenafil have been proposed to improve pulmonary vasodilation. In this way, the novelty of this work lies in assessing the effect of these treatments in a relatively understudied artery in the context of hypobaric hypoxia as the aorta, through biomechanics and histomorphometry. In this regard, only two studies have investigated the impact of hypobaric hypoxia on vascular biomechanics (Aguilar et al., 2018; Utrera et al., 2022). Additionally, two other studies (Navarrete et al., 2020; Rivera et al., 2020) examined the systemic circulation level effects of drug treatments such as melatonin, atrial natriuretic peptide (ANP), and cinaciguat. All the mentioned works have been developed by our research group, and the current study aligns with them, providing further insight into this condition and its cardiovascular implications.

The experimental setup presented in this work enables us to postulate the existence of a relationship between physiological strain and mechanical properties in the vessel with its corresponding stress state. In this context, the seminal work by Cardamone et al. (2009) stated that longitudinal pre-stress and circumferential residual stress on arteries are contributions to their adaptation mechanisms in the face of perturbations triggered by changes in the external conditions with the aim at maintaining a homeostatic state, and hence its clinical importance since its levels are altered in the face of change in the environment (injuries and diseases) (Sigaeva et al., 2019). Therefore, assessing the level of physiological non-loaded stress distribution in the arterial wall and its possible variation in response to treatments is essential to clarify the contribution of the latter to the mitigation of the adverse effects of a HAHH condition.

**FIGURE 6:**
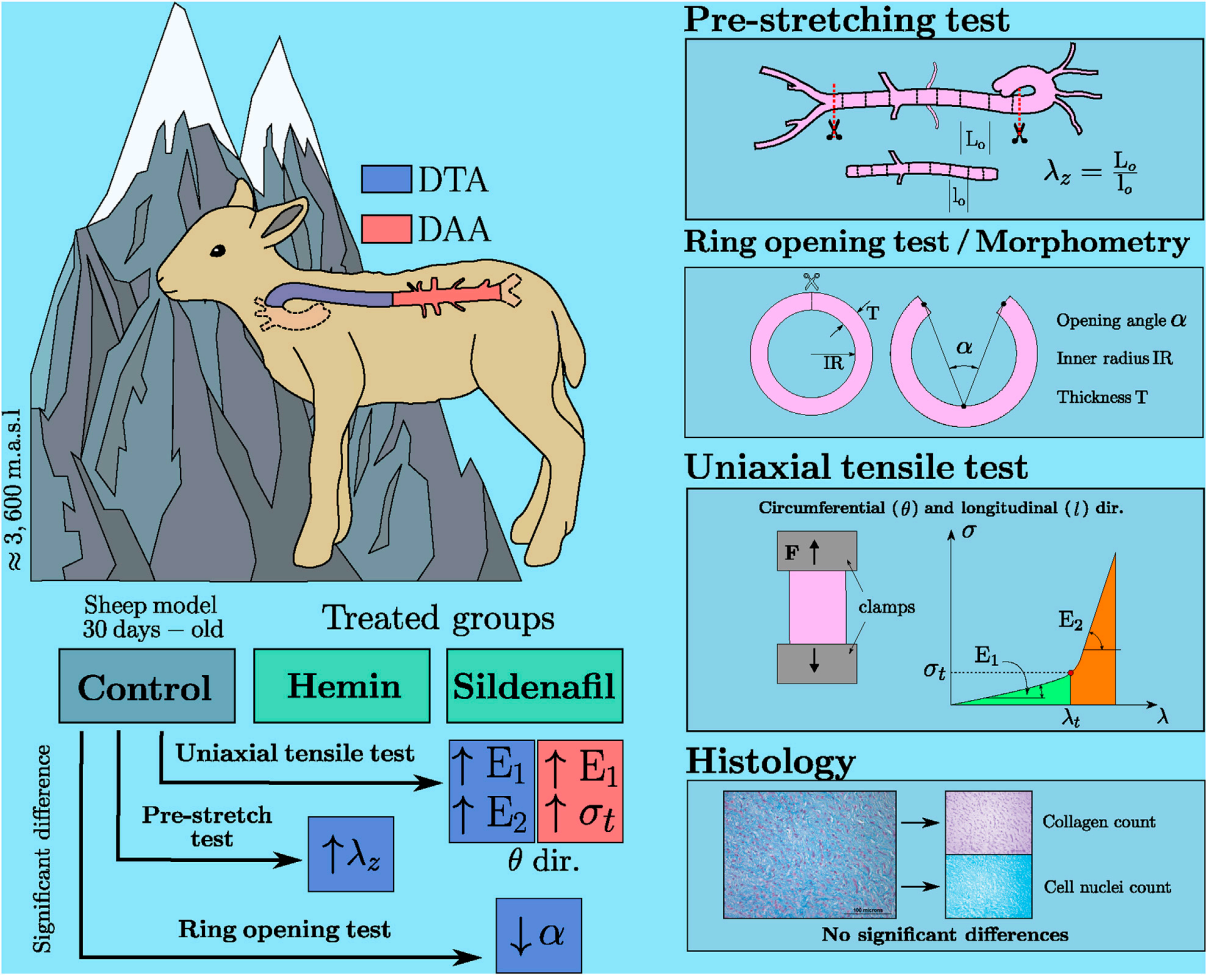
Main results of biomechanical and morpho-structural analysis of hemin/sildenafil treated groups. Representative scheme.

Deepening in the main findings related to this work, that are also summarized in Figure 6, they exhibit that hemin and sildenafil treatments affect the biomechanics of the aorta, with a significant increment in pre-stretch values of DTA in animals treated with hemin, and a decrement in opening-angle value of DTA along with a higher uniaxial stiffness along the circumferential direction of DTA and DAA, due to a sildenafil treatment.

Related previous works report similar axial stretch evolution $\left(\lambda_{Z}\right)$ from proximal towards distal zones of the aorta, with comparable values for each section. Rivera et al. (2020) determined pre-stretch levels under the same protocols and animal-model used in this work, on individuals treated with melatonin. Their main findings determined an incremental variation of 1.1–1.5 approximately, going from the thoracic to the abdominal aorta, in the same way as shown in Figure 3. Under the same chronic-hypobaric hypoxia condition, Navarrete et al. (2020) established $\lambda_{Z}$ values in different sections of the aorta of neonatal lambs (15 days-old) treated with vasodilator drugs ANP and Cinaciguat. Their results show a similar evolution to those shown in Figure 3, with $\lambda_{Z}$ values ranged between 1.1 and 1.6 approximately. According to Navarrete et al. (2020) and Rivera et al. (2020), treated groups did not exhibit significant changes in $\lambda_{Z}$ values along the aorta compared to the control group. In addition to the aforementioned works, the study of Utrera et al. (2022), applied in a context of intermittent hypobaric hypoxia on rats, showed a statistically-significant change of pre-stretching values on the aorta between groups subjected to normobaric normoxia, short-term and long-term intermittent hypobaric hypoxia. Other research in this area addressed axial pre-stretch levels in the face of different variables, such as age (Horny et al., 2014b), type of vessel and its respective size (Guo et al., 2012), type of artery (Horny et al., 2017), and the effect of atherosclerosis and post-mortem interval on human abdominal arteries (Horny et al., 2014a). However, the effect of a hemin treatment on $\lambda_{Z}$ values has not been previously reported in studies assessing the effects of hypobaric hypoxia. Delving into the effects associated with the physiological role of axial pre-stretch $\lambda_{\text{Z}}$ , there is evidence that higher values of this parameter are related to the homogenization in the longitudinal stresses and the energy conservation by each pressure cycle, a higher degree of arterial distensibility, and prevention of buckling (Cardamone et al., 2009; Horny et al., 2014b; García-Herrera et al., 2016; Kamenskiy et al., 2016). Therefore, the administration of hemin increased the level of physiological axial stretch reported in DTA, indicating an improvement in vasodilation performance. In addition, an increase in $\lambda_{Z}$ by itself is also associated with higher levels of longitudinal pre-stress.

Regarding the ring opening angle behavior in the context of chronic hypobaric hypoxia, a similar behavior is observed in related works (Herrera et al., 2008; Navarrete et al., 2020; Rivera et al., 2020). In these studies, DTA zones presented opening-angle values ranged between 80 ° and 140 ° approximately, whereas in the DAA region values declined, ranging between 40 ° and 60 ° approximately. In specific, Navarrete et al. (2020) found a significant decrement of this value due to ANP and Cinaciguat treatments, in a transition zone between DTA and DAA. This same trend was also reported in other cardiovascular regions, such as carotid arteries. However, according to Rivera et al. (2020), melatonin treatment did not affect the aortic zones (DTA → 140 °± 16 ° ; DAA → 48 °± 9 ° ). In addition, it is worth stating that there is also a dependence between the animal model studied, the external condition to which specimens are exposed, and the characteristic mean opening angle value. For instance, in thoracic aortas of rats exposed to intermittent hypobaric hypoxia (Utrera et al., 2022), mean opening angles were found close to 40 ° , while at a sea-level condition this value was close to 100 ° . Moreover, in studies performed on porcine aortas (Sokolis et al., 2020), the opening angle presented variations among artery layers. On the other hand, García-Herrera et al. (2016) described variations of the opening-angle in the face of diseases, such as Marfan syndrome, aneurysms and bicuspid valve on human ascending thoracic aortas. Providing an interpretation about the opening angle in the face of sildenafil treatment for DTA (Figure 3), a lower value of this parameter by itself (keeping all other conditions constant) is indicative of a lower degree of residual stress in the arterial wall, which accomplishes a homogenizing role of the transmural stress distribution in the *in-vivo* state (Cardamone et al., 2009).

There have been previous studies aimed at studying the biomechanical response of arteries subjected to HAHH which included the analysis of uniaxial-tensile tests. Among the main findings reported by related works, Utrera et al. (2022) performed uniaxial-tensile tests only along the longitudinal direction on samples of aorta arteries of rats exposed to acute and chronic intermittent hypoxia (low and high number of HAHH cycles, respectively) without a pharmacological treatment, finding significant differences only on the slope at high-stretches $E_{2}$ between animals subjected to acute and chronic hypoxia conditions. Likewise, Aguilar et al. (2018) performed uniaxial-tensile tests on femoral arteries of rats subjected to intermittent hypoxia, using ring-shaped samples instead of the prismatic samples considered in this work. The initial and final slopes of stress-stretch curves (mechanical properties, $E_{1}$ and $E_{2}$ , respectively) were compared. Main findings set significant differences on the $E_{2}$ parameter, which increased under hypoxic conditions. On the other hand, Rivera et al. (2020) performed uniaxial-tensile tests on main pulmonary, thoracic and abdominal aorta arteries from lambs subjected to chronic HAHH, evaluating a possible alteration due to treatment with melatonin. Therein, no significant differences were found on the mechanical parameters from uniaxial-tensile tests, neither on slopes at low and high strain, nor on the transition stress and stretch. Likewise, Bezmalinovic et al. (2021) evaluated thoracic aortas of lambs subjected to chronic HAHH and treated with melatonin, finding no significant differences on the uniaxial stress. After analyzing the biomechanical parameters presented in Tables 2, 3, it can be seen that there is a tendency for a greater degree of material stiffness in both arterial zones when treatment is applied. However, it becomes significant only in those experimental groups treated with sildenafil in the circumferential direction of the vessel. It should be noted that the increase in this parameter by itself reflects an increment in the circumferential residual stress levels.

Coupling the effect of the biomechanical results discussed above, it is possible to determine that sildenafil treatment triggers an alteration in the biomechanical properties of DTA and DAA, where a stiffness increment along the circumferential direction is reported in both zones (Tables 2, 3). Particularly for DTA, although higher values of the circumferential stiffness at low stretches $E_{1}$ (a parameter representative of the physiological conditions of the artery) are exhibited, the fact that the residual strain (represented by the opening angle) in this direction was lower, it does not provide enough evidence to back up a potential change on the residual stress. Pertaining DAA zones, from Table 3 and Figure 4 it is possible to underline that arteries treated with sildenafil had a higher residual stress along the circumferential direction, due to differences in mechanical properties $E_{1}$ and $\sigma_{t}$ , whereas the opening angle did not exhibit differences due to this treatment. As for the mechanical parameters and pre-stretch found along the longitudinal direction of DTA zones, results of the hemin-treated group and its comparison with the Control group (see Figure 3 and Table 2) show that there were no significant differences on the mechanical parameters. However, axial pre-stretch levels were significantly higher in the treated group, when compared to the Control group. Therefore, it can be stated that the axial pre-stress level on DTA was higher for the hemin-treated group. Since all other group-wise comparisons lacked significant differences in mechanical properties and residual strains, no evidence supports changes in residual stresses in these cases.

In summary, all biomechanical differences reported between groups indicate a stress level increment in treated groups: (a) physiological axial pre-stress for hemin in DTA, and (b) circumferential residual stress for sildenafil in DAA. This increase in residual stress might be interpreted as a homogenizing effect on the arterial wall (Haghighipour et al., 2010). In this way, the transmural stress distribution tends to be more uniform when compared to that of the hypoxic non-treated group. Beñaldo et al. (2022) report the systemic arterial pressure in animals exposed to both treated and untreated hypoxia. The values remain relatively constant throughout the perinatal period, indicating that our results are not affected by blood pressure variations when treatments are administered. Therefore, changes in the unloaded physiological stress induce a positive biomechanical response in groups treated with hemin and sildenafil.

Arteries are structured with three main layers: the intima, media, and adventitia. The intima comprises a sole layer of endothelial cells resting on a delicate extracellular matrix. The media contains multiple layers of smooth muscle cells capable of producing various structural components like collagen, elastin, proteoglycans, and growth factors. The coordinated actions of endothelial and smooth muscle cells play a crucial role in regulating responses to hemodynamic and biochemical cues, influencing both normal development and physiological as well as pathological reactions (Kumar et al., 2014). Smooth muscle cells within blood vessel walls are linked to a complex network of collagen fibers and elastic fibers. This network plays a crucial role in allowing the vessel wall to stretch elastically, enabling it to accommodate the volume of blood ejected during each heartbeat and to dampen pressure fluctuations in the periphery. The activity and arrangement of smooth muscle cells can impact the mechanical stress experienced by the extracellular matrix components, thereby regulating vessel diameter and stiffness (Holzapfel, 2008; Reesink and Spronck, 2019). Focusing on histological results, in general terms, the collagen content generally aligns with findings from previous studies. Specifically, for healthy sheep under 18 months old Graham et al. (2011) report a collagen percentage close to 30 % In other animal models such as mini-pigs, collagen percentage ranges between 30 and 50 % approximately in the first 8 weeks of life Hayenga et al. (2012). In addition, Concannon et al. (2020) set the collagen amount in the human thoracic aorta via Masson’s Trichrome stain, as between 20 and 35 % . Cell nuclei density has been also determined in different research. Wheeler et al. (2015) using a mouse animal model, report an average value close to 4,000 cells/ ${mm}^{2}$ in young rodents. In the aformentioned work of Concannon et al. (2020) approximately 1,000 cell nuclei by ${mm}^{2}$ are reported in the human thoracic aorta. Utrera et al. (2022) exhibit cell nuclei density values around 2000 cells/ ${mm}^{2}$ in the thoracic aorta of normoxic and (intermittent) hypobaric-hypoxic rats. Finally, Rivera et al. (2020) determine values close to 3,000 cells/ ${mm}^{2}$ for hypoxic sheep.

O’Rourke and Nichols (2005), it was demonstrated that chronic exposure to arterial hypertension results in increased stress on smooth muscle cells, prompting them to proliferate. This proliferation leads to hyperplasia (increased cell numbers) and thickening of the vascular wall (Jaminon et al., 2019). Additionally, Humphrey et al. (2014) showed that matrix metalloproteinase activity facilitates the breakdown of elastin within the extracellular matrix. In response to this breakdown, synthetic smooth muscle cells begin producing collagen ECM in an attempt to maintain stiffness homeostasis. Considering these findings, the lack of significant variations in collagen density and cellular nuclei count within the arterial walls between the untreated and treated groups suggests that the administered drugs do not have an effect on cell density but do not discard a possible effect on smooth muscle cell function or other structural components that may affect the biomechanical characteristics or vasoactive function.

As depicted in Figure 5, there is a lack of histological evidence in DTA supporting the notion that the biomechanical modifications outlined in this study can be attributed to the proliferation or decrease of microstructural load-bearing components or alterations at macrostructural levels, as illustrated in Table 1. To address this pertinent issue, it is conceivable to attribute the overall mechanical alteration expounded upon in Section 3.2 to changes in the stress/strain state of extracellular matrix components throughout the aortic-wall lifespan. This perspective finds resonance in prior investigations (Zeller and Skalak, 1998; Humphrey and Rajagopal, 2002).

All the mentioned aspects highlight that arterial biomechanics emerges as a crucial approach in explaining cardiovascular functional changes in response to hypobaric hypoxia, as well as the potential for improvement through treatment interventions (Herrera et al., 2008). This notion becomes particularly valuable, given that in the case study of this research, histological and morphometric information by itself does not provide evidence about the beneficial effects of treatment application, as reported in the biomechanical analysis.

A critical issue to be addressed in the biomedical research field is the potential translation of the results obtained from the sheep animal model to humans. Several authors (Duchenne et al., 2019; Weisskopf et al., 2021; Banstola and Reynolds, 2022) have discussed the impact of the sheep model in a cardiovascular context, assessing the following advantages: (a) Similar to cardiovascular human anatomy and physiological functions; (b) adequate translation of drug therapy between pre-clinical to clinical studies; (c) similar development with human pregnancy; and (d) biomechanical and hemodynamic properties are comparable to humans. In addition, focusing the attention on studies contextualized on perinatal chronic hypoxia using the ovine animal model, Gonzaléz-Candia et al. (2020) highlight the importance and benefits of this animal model, particularly on the translation to cardiopulmonary development. Moreover, Morrison et al. (2018) refers to the importance of the use of the sheep model in the study of intrauterine fetal growth restriction through high-altitude pregnancy, since the development of this condition is similar to humans, compared to other frequent-used animal models.

On one hand, the strength of this study lies in the biomechanical analysis of the effect of drug-based treatments on the passive biomechanical response of arterial tissue performed through multiple approaches, namely, *ex-vivo* mechanical testing along with morphological and histological analysis. This allows us to link the mechanical response of the arterial wall with the geometry and microstructure of the extracellular matrix and smooth muscle cells (Gleason and Humphrey, 2004). The study was performed on an appropriate animal model, whose results can be translated to clinical trials. On the other hand, the limitations of this study are related to, firstly, having analyzed only the passive response of arterial tissue, and secondly, the morphometric and histological analysis of the arterial wall, performed by accounting for microstructural components evaluated only on the transversal section of the arterial wall.

## 5 Conclusion

In this work, the biomechanical response of descending-thoracic aortas (DTA) and distal-abdominal aortas (DAA) from lambs subjected to chronic hypobaric high-altitude hypoxia (HAHH) has been studied, assessing whether hemin and sildenafil treatments could alter this behavior.

Our findings show that both treatments affect the biomechanics of the aorta, marked by: a significant increase in pre-stretch values on DTA subjects treated with hemin, which has not been reported in previous works; added to a decrement in opening-angle values on DTA and to higher uniaxial stiffness along the circumferential direction on DTA and DAA, due to sildenafil treatment. These results bring as a consequence an increment in stress level for an unloaded *in-vivo* configuration of the treated groups, compared to the non-treated, which improves vasodilatation, along with homogenizing and diminishing the stress levels on the artery wall, both desired effects concerning the application of the treatments.

Our results allow us to elucidate the impact of sildenafil and hemin treatments on the axial pre-stress and the circumferential residual stress of arteries from lambs exposed to HAHH, granting some insight into the relationship between the functional improvement of these pharmacological treatments and arterial biomechanics. In addition, the importance of the choice of the animal model is emphasized, determining that the sheep animal model is feasible to reduce the gap between this pre-clinical study and the clinical application of these findings.

To deepen the knowledge about the biomechanical response of the artery wall, future investigations should be focused on the study of other phenomena, such as viscoelasticity and active response. Along with that, adopting a feasible method to obtain the transmural residual stress (e.g., via numerical simulations) could give discernment about the quantitative impact of drug-based treatments directed at mitigating the effects of exposure to high altitudes, relating them to potential remodeling phenomena triggered at the cellular level. In addition, future studies should focus on the utilization of novel experimental techniques, such as confocal microscopy and computer tomography. These techniques enable precise quantification of fiber configurations under various load states (Pukaluk et al., 2022) and their spatial disposition along different cross-sectional views of the blood vessel. This approach aims to discern, more accurately, whether biomechanical changes stem from alterations in arterial components rather than their relative quantity.

## Data Availability

The raw data supporting the conclusions of this article will be made available by the authors, without undue reservation.
